# Single-center experience with levosimendan in children undergoing cardiac surgery and in children with decompensated heart failure

**DOI:** 10.1186/1471-2253-11-18

**Published:** 2011-10-05

**Authors:** Pertti K Suominen

**Affiliations:** 1 Department of Anesthesia and Intensive Care, Children's Hospital, Helsinki University, Central Hospital, P.O B. 281 Stenbäckinkatu 11, FIN-00029 HUCS Helsinki, Finland

## Abstract

**Background:**

Levosimendan has pharmacologic and hemodynamic advantages over conventional intravenous inotropic agents. It has been used mainly as a rescue drug in the pediatric intensive care unit or in the operating room. We present the largest single-center experience of levosimendan in children.

**Methods:**

Retrospective analysis of all children who received levosimendan infusions between July 5, 2001 and July 4, 2010 in a pediatric intensive care unit. The results of a questionnaire for physicians (anesthesiologist/intensivists, cardiologists and cardiac surgeons) concerning their clinical perceptions of levosimendan are evaluated

**Results:**

During the study period a total of 484 infusions were delivered to 293 patients 53% of whom were male. The median age of the patients was 0.4 years (4 hours-21.1 years) at the time of levosimendan administration. A majority of levosimendan infusions were administered to children who were undergoing cardiac surgery (72%), 14% to children with cardiomyopathy and 14% to children with cardiac failure. Eighty-nine out of the 293 patients (30.4%) received repeated doses of levosimendan (up to 11 infusions). The most common indication for the use of levosimendan (94%) was when the other inotropic agents were insufficient to maintain stable hemodynamics. Levosimendan was especially used in children with cardiomyopathy (100%) or with low cardiac output syndrome (94%). A majority (89%) of the respondents believed that levosimendan administration postponed the need for mechanical assist devices in some children with cardiomyopathy. Moreover, 44% of respondents thought that the mechanical support was totally avoided in some patients undergoing cardiac surgery after receiving levosimendan.

**Conclusion:**

Levosimendan is widely used in our institution and many physicians believe that its use could decrease the need for mechanical support in children undergoing cardiac surgery or in children with decompensated heart failure. However, there is a lack of good empirical evidence in children to support this perception.

## Background

Levosimendan was developed for the treatment of decompensated heart failure in situations for which conventional therapy is not sufficient [1]. Levosimendan acts independently of β-adrenergic receptors and cyclic adenosine monophosphate (cAMP). Therefore it has pharmacologic and hemodynamic advantages over conventional intravenous inotropic agents [1,2]. It exerts its effect mainly through two different mechanisms. The first mechanism is by calcium sensitization of the contractile proteins in the cardiomyocytes, which leads to increased contractility of the heart [1,2]. The second mechanism opens the adenosine triphosphate-sensitive potassium channels on vascular smooth muscle, which causes coronary and peripheral vasodilatation [1,2].

Levosimendan has a short half-life of approximately one hour but its action has a fast onset and long-lasting due to the active metabolite OR-1896 [2,3]. The maximum concentration of OR-1896 can be detected two days after the 24-hour levosimendan infusion has stopped, and its elimination half-life is 70-80 hours in patients with heart failure [2].

Levosimendan appears to be efficacious and safe for children with acute heart failure or children who are undergoing cardiac surgery [3-11]. The number of children in the retrospective and mainly observational studies has not been extensive as the largest dataset included 19 patients [3,11]. Levosimendan has been used "off-label" as a rescue drug in the pediatric intensive care unit (PICU) or in the operating room (OR), because there are no official indications for its use in patients under 18-years of age to date.

In our institution levosimendan has been used in children with decompensated heart failure and in children undergoing cardiac surgery since 2001. The aim of this review is to present retrospective data on levosimendan administration and physicians surveyed for their perceptions about levosimendan's efficacy and safety.

## Methods

This is a retrospective analysis of data obtained from the patients who received levosimendan infusion between July 5, 2001 and July 4, 2010 in the PICU of Children's Hospital of Helsinki University Central Hospital. The Children's Hospital is a tertiary care teaching hospital responsible for the care of all paediatric patients, in Finland (population 5.4 million) who *inter alia *require open-heart surgery and transplantation.. This study was approved by the Ethics Committee of Helsinki University Central Hospital.

Information regarding the levosimendan administration was collected from the anaesthesia records and the intensive care database (Centricity Critical Care Clinisoft; GE Healthcare, Helsinki, Finland). The patients who received levosimendan were classified into one of three groups namely: cardiac surgery, cardiomyopathy and cardiac failure. The cardiac surgery group included patients to whom levosimendan infusions were administered pre- peri or postoperative during the same PICU admission in which they had surgery. The cardiac failure group comprised patients with decompensated cardiac failure who had not been operated on during the PICU admission. These patients also had cardiac failure whose etiology was exclusive of cardiomyopathy. The third group consisted of children with dilated cardiomyopathy, who had been admitted to the PICU because of acute or chronic heart failure to receive a repeated dose of levosimendan. Cardiomyopathy patients on a left sided mechanical assist device (Berlin Heart) who received levosimendan to support the right ventricle, or had cardiac transplantion during the PICU admission were also included in the cardiomyopathy group.

Each patient was included in only one of the three patient groups in any one of the nine study years. Therefore, a patient who had cardiac surgery and was re-admitted to the PICU during that same year was still considered to be a surgical patient. However, if a child was admitted to the PICU several times over different years, it was counted as a separate patient. Repeated infusions of levosimendan were calculated on a yearly basis between January 1 and December 31, unless the same PICU admission continued into the following year.

Levosimendan infusion was usually started with a bolus dose of 12 to 24 mcg/kg over 10 min followed by 0.1 mcg/kg/min infusion. The dose was increased slowly to 0.2 mcg/kg/min and usually the total duration of the infusion was 24 hours, but in some patients infusion was continued for up to 48 hours. The bolus dose was omitted in some of the unstable hypotensive patients as recommended in the adult literature [12]. Levosimendan was coadministered in different combinations with the other vasoactive drugs including: epinephrine, norepinephrine, milrinone, vasopressin and inhaled nitric oxide.

A survey of physicians was carried out by questionnaire at the end of August 2010, before the results of the levosimendan audit in our hospital were presented by the author. A questionnaire was sent via email to all the specialists of anaesthesiology, cardiology and cardiac surgery (Additional file 1). The questionnaire consisted of 10 multiple choice questions and was specially designed to determine the indications, patient groups, possible adverse events and perceptions of the effects of levosimendan infusions the physicians could recall.

## Results

During the nine year study period a total of 484 infusions were administered to 293 patients during 369 PICU admissions. The median age of the children at the time of levosimendan administration was 0.4 years (range: 4 hours - 21.1 years). Moreover, 65% of the patients were younger than one year of age (Table 1). A small majority of the patients were boys 197 (53.3%).

**Table 1:** Ages of the children who received levosimendan infusion during 369 PICU admissions

Age	Number	Percentage
<1 months	89	24.12
1-12 months	151	40.92
1-4 years	73	19.78
5-12 years	31	8.40
>12 years	25	6.78
Median (year)	0.40	
Interquartile range (year)	0.09- 2.08	
Range	4 hours - 21.1 years	

A majority of levosimendan infusions were administered to children undergoing cardiac surgery (n = 210, 72%) and 14% of levosimendan infusions were respectively given to children either with cardiomyopathy (n = 41) or who had decompensated heart failure (n = 42). In the cardiomyopathy group 36 patients had dilated, two restrictive, one obstructive cardiomyopathy and in two patients the etiology was undefined. Of those children with decompensated heart failure, 32 had congenital heart disease that had not been operated on during the PICU admission, five had pulmonary hypertension, two had arrhythmias and three had other etiologies.

**Figure 1:**
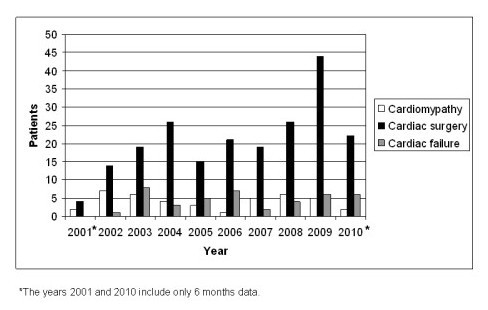
**The etiology of 293 patients administered with levosimendan infusion**.

The annual increase in levosimendan use was related to cardiac surgery patients (Figure 1). Of all the 331 infusions given to children undergoing cardiac surgery, 25% were initiated in the OR. A trend towards the initiation of levosimendan infusion before surgery in the PICU or after induction of anesthesia has occurred in recent years. The most common defects in children who had undergone cardiac surgery and who received levosimendan, are shown in table 2. Twenty of the 36 patients with hypoplastic left heart syndrome (55.6%) were neonates undergoing the Norwood 1 operation.

**Table 2:** The most common etiologies of the 210 children who were undergoing congenital heart surgery who received levosimendan infusions either in the OR or in the PICU

	Number
Hypoplastic left heart syndrome	36
Transposition of great arteries	24
Atrioventricular septal defect	18
Univentricular heart (other)	15
Aortic stenosis	13
Pulmonary atresia	11
Ventricular septal defect	11
Tetralogy of Fallot	10
Interrupted aortic arch	9
Truncus arteriosus	8
ALCAPA	7
Double outlet right ventricle	7
Coarctation of the aorta	7
Ebstein anomaly	6
other	28

ALCAPA, anomalous left coronary artery from pulmonary artery.

Eighty-nine out of the 293 patients (30.4%) received repeated doses of levosimendan (up to 11 infusions). Of the repeated doses 66.3% were administered to cardiac surgery patients, 24.7% to cardiomyopathy and 9.0% to cardiac failure patients. The median dosing interval was 9.1 days (range 2.7- 264.1 days).

During the nine year study period, 26 patients (0.9% of the total number of operations.n = 2566) required mechanical cardiopulmonary support in the postoperative period. All except one of the 24 patients [16 patients in left or right ventricular assist device (LVAD, RVAD), seven in extracorporeal membrane oxygenation (ECMO) and three in intraaortic balloon pump (IABP)] received levosimendan before or after placement onto the mechanical assist device to enhance weaning from the device or support the right ventricle during the support period. Seventeen of the 24 patients (70.1%) were successfully weaned from the device. Of the patients with cardiomyopathy one patient was treated by ECMO, one by LVAD and eight by a long term support device Berlin Heart VAD. All of them also received levosimendan infusion during the mechanical support. Six of the eight patients with cardiomyopathy were successfully bridged to heart transplantation after seven to 189 days support by the Berlin Heart VAD.

Of the 293 children who received levosimendan infusions, 36 (12.4%) died in the PICU. Twenty-two (10.5%) children of the cardiac surgery group died compared to the overall mortality of 2.3% for the children undergoing cardiac surgery during the same study period.

Eighteen of the 29 (62.1%) anesthesiologist/intensivists, cardiologists and cardiac surgeons to whom the survey was emailed responded. There were no differences in the answers from the different specialists. Levosimendan was considered to be safe and efficacious for use in children based on the extensive experimental and adult patient studies by 88.9% of the respondents. However, it was generally considered that there was not enough evidence from the few existing studies done in children to warrant the routine use of levosimendan. The most common indication for the use of levosimendan (94%) reported by the respondents was when other inotropic agents were insufficient to maintain stable hemodynamics. Levosimendan was especially used in children with cardiomyopathy (100%) or with low cardiac output syndrome (94%). Levosimendan infusions were well tolerated, only hypotension (62.1%) or tachycardia (27.8%) occurred in the beginning of the infusion or no adverse events (27.8%) were recalled by the physicians. According to the survey 89% of the physicians thought that levosimendan administration had postponed the need for the mechanical assist device in some children with dilated cardiomyopathy. Moreover, 44% of respondents thought that the mechanical support had been totally avoided in some patients undergoing cardiac surgery after receiving levosimendan. Eleven out of 18 physicians (61.1%) thought that levosimendan treatment had saved the lives of some children when the other treatments had failed.

## Discussion

Levosimendan is the best investigated inotropic drug in adults with more controlled clinical data for it than any other inotropic drug [1]. However, reported pediatric experience and randomized levosimendan trials in children are few in number [3,11]. This study is the largest published study on levosimendan use in children to date, with data on 484 levosimendan infusions delivered to 293 patients. The perioperative use of levosimendan had been steadily increasing over the years. The prevalent perceptions of the physicians who responded were that levosimendan may have postponed or have reduced the need of mechanical cardiac support in children with cardiomyopathy or who were undergoing cardiac surgery. Nevertheless, this perception remains to be proven in randomized controlled studies.

There is no unequivocal answer as to why our institution uses levosimendan more widely than in other comparable pediatric cardiac centers. Similar to that described in some case reports published, we have had some miraculous recoveries of patients after the initiation of levosimendan infusion [10,11]. Based on some preliminary studies in our institution and mostly involving adults, the administration of levosimendan has become one of the standard regimes in children with: failing ventricular function or for proactive use to facilitate weaning from cardiopulmonary bypass in children who are undergoing cardiac surgery [3,13]. Levosimendan has been included in the expensive drugs list in our hospital, but has been readily available when indicated without any special authorization.

Levosimendan use in children with cardiomyopathy or decompensated failure remained at the same level during the nine year period. The observed increase in levosimendan use was related to cardiac surgery patients. A trend towards the initiation of levosimendan infusion in the PICU before surgery or after induction of anesthesia has occurred in recent years. This trend is based on the adult cardiac surgery experience that the full clinical potential of levosimendan is best realized when it is used in a prophylactic manner: i.e. started before CPB commences and before the onset of surgical ischemia in patients at risk of developing low-output syndrome after CPB [12,14,15]. In the only randomised and double-blind trial in children undergoing cardiac surgery, levosimendan was shown to be as efficacious as milrinone, although, levosimendan was started after the release of the aortic cross clamp with a relatively low dose 0.05 mcg/kg/min and without an initial bolus dose [6].

During the nine year study period the use of postcardiotomy mechanical support (0.9%) was fairly low in our institution compared to that reported in the pediatric literature [16]. In our institution levosimendan was initiated proactively in the OR or when the weaning from CPB failed at the first attempt for many of the higher risk cases. Most likely the threshold as to when to switch from inotropic support to mechanical support is higher than in many other pediatric cardiac centers. Therefore, it is impossible to conclude whether levosimendan treatment had any effect on the use of postcardiotomy mechanical support.

Mortality in children who received levosimendan infusions after cardiac surgery (11%) was higher than the overall mortality of 2.3% after cardiac surgery during the same study period. Levosimendan was mainly administered to the high risk patients and after the other inotropic agents were found to be insufficient to maintain stable hemodynamics. Therefore, a higher mortality rate similar to that found for earlier pediatric levosimendan studies including those on high risk patients was to be expected [4,7,9].

The perception shared by many respondents, that repeated levosimendan infusions may have postponed the need of mechanical cardiac support in children with cardiomyopathy, remains to be proven in future studies. At the moment we have no data on the effects of repeated levosimendan infusions on the ejection fraction or any laboratory parameters such as brain natriuretic peptide (BNP) in patients with cardiomyopathy. In the placebo control study carried out by Parissis et al. serial levosimendan infusion of adult patients with decompensated heart failure significantly improved the left ventricular performance and decreased BNP and immune activation [17].

An important shortcoming of our study is that it is a retrospective study. The number of physicians who responded to the questionnaire (62%) was somewhat low, because many were still on summer vacation during the survey and consequently never completed it. We did not use any specific risk classification for patients in cardiac failure. Therefore levosimendan infusion was started based on the clinical findings of the cardiac failure patient including poor hemodynamic profile, metabolic acidosis, poor tissue perfusion, oliguria and rising serum lactate levels despite escalating inotropic support. The information regarding other vasoactive drugs that these patients were receiving prior to introduction of levosimendan and information on the impact of levosimendan on discontinuation of other vasoactive drugs, would have been extremely informative. Unfortunately, the patient data in this study are heterogeneous, and indications and timing about when to use levosimendan has been variable. There is a lack of randomized controlled studies on the use of levosimendan in children, thus our clinical practice and perceptions of the effects of levosimendan on the clinical outcome serves as a good example of institutional based medicine rather than empirical evidence based medicine.

## Conclusions

In the institution in which levosimendan is widely used, many physicians believe that its use could have an effect on the extent of mechanical support needed in children undergoing cardiac surgery or decompensated heart failure, although there is still a lack of good evidence to support this perception. Randomized and controlled studies on the use of levosimendan in children are warranted.

## Competing interests

Pertti Suominen has received two modest speaker fees from Orion (Levosimendan).

## Authors' contributions

PKS did the data analysis, interpretation, writing of the manuscript and approved the final manuscript.

## Pre-publication history

The pre-publication history for this paper can be accessed here:

http://www.biomedcentral.com/1471-2253/11/18/prepub

## Supplementary Material

Additional file 1 **Levosimendan questionnaire**. A multiple choice questionnaire for physicians. Click here for file

## References

[B1] De Luca L Colucci WS Nieminen MS Maissie BM Gheorghiade M Evidence-based use of levosimendan in different clinical settings Eur Heart J 2006 27 1908 1920 10.1093/eurheartj/ehi875 16682381

[B2] Antila S Sundberg S Lehtonen LA Clinical pharmacology of levosimendan Clin Pharmacokinet 2007 46 535 552 10.2165/00003088-200746070-00001 17596101

[B3] Turanlahti M Boldt T Palkama T Antila S Lehtonen L Pesonen E Pharmacokinetics of levosimendan in pediatric patients evaluated for cardiac surgery Pediatr Crit Care Med 2004 5 457 462 10.1097/01.PCC.0000137355.01277.9C 15329162

[B4] Namachivayam P Crossland DS Butt WW Shekerdemian LS Early experience with levosimendan in children with ventricular dysfunction Pediatr Crit Care Med 2006 7 445 448 10.1097/01.PCC.0000235251.14491.75 16885788

[B5] Magliola R Moreno G Vassallo JC Landry LM Althabe M Balestrini M Charroqui A Salgado G Lataza E Chang AC Levosimendan, a new inotropic drug: experience in children with acute heart failure Arch Argent Pediatr 2009 107 139 145 19452086 10.1590/S0325-00752009000200008

[B6] Momeni M Rubay J Matta A Rennotte MT Veyckemans F Poncelet AJ Clement de Clety S Anslot C Joomye R Detaille T Levosimendan in congenital cardiac surgery: A randomized, douple-blind clinical trial J Cardiothorac Vasc Anesth 2011 25 419 24 10.1053/j.jvca.2010.07.004 20829069

[B7] Garisto C Favia I Ricci Z Di Chiara L Morelli S Giorni C Vitale V Picardo S Di Donato RM Initial single-center experience with levosimendan infusion for perioperative management of univentricular heart with ductal-dependent systemic circulation World J of Pediatr and Congenital Heart Surg 2010 1 292 299 10.1177/2150135110378310 23804885

[B8] Osthaus WA Boethig D Winterhalter M Huber D Goerler H Sasse M Sűmpelmann First experiences with intraoperative Levosimendan in pediatric cardiac surgery Eur J Pediatr 2009 168 735 740 10.1007/s00431-008-0834-7 18813947

[B9] Egan JR Clarke AJB Williams S Cole AD Ayer J Jacobe S Chard RB Winlaw DS Levosimendan for low cardiac output: a pediatric experience J Intensive Care Med 2006 21 183 187 10.1177/0885066606287039 16672640

[B10] Lechner E Moosbauer W Pinter M Mair R Tulzer G Use of levosimendan, a new inodilator, for postoperative myocardial stunning in a premature newborn Pediatr Crit Care Med 2007 8 61 63 10.1097/01.PCC.0000253026.67341.5D 17149153

[B11] Braun J-P Schneider M Kastrup M Liu J Treatment of acute heart failure in infant after cardiac surgery using levosimendan Eur J of Cardiothoracic Surg 2004 26 228 230 10.1016/j.ejcts.2004.03.034 15201012

[B12] Salmenperä M Eriksson H Levosimenendan in perioperative and critical care patients Curr Opin in Anaesthesiol 2009 22 496 501 10.1097/ACO.0b013e32832c5269 19502977

[B13] Turalahti M Mildh L Peltola K Rautiainen P Initial experience of levosimendan in pediatric patients Pediatr Cardiol 2004 25 603

[B14] Eriksson HI Jalonen JR Heikkinen LO Kivikko M Laine M Leino KA Kuitunen AH Kuttila KT Peräkylä TK Sarapohja T Suojaranta-Ylinen RT Valtonen M Salmenperä MT Levosimendan facilitates weaning from the cardiopulmonary bypass in patients undergoing coronary artery bypass grafting with impaired left ventricular function Ann Thorax Surg 2009 87 448 454 10.1016/j.athoracsur.2008.10.029 19161758

[B15] Landoni G Mizzi A Biondi-Zoccai G Bruno G Bignami E Corno L Zambon M Gerli C Zangrillo A Reducing mortality in cardiac surgery with levosimendan: A meta-analysis of randomized controlled trials J Cardiothorac Vasc Anesth 2010 24 51 57 10.1053/j.jvca.2009.05.031 19700350

[B16] Chang AC McKenzie ED Mechanical cardiopulmonary support in children and young adults: Extracorporeal membrane oxygenation, left ventricular assist devices and long-term support devices Pediatr Cardiol 2005 26 2 28 10.1007/s00246-004-0715-4 15156301

[B17] Parissis JT Adamopoulos S Farmakis D Filippatos G Paraskevaidis I Panou F Iliodromotis E Kremastinos DT Effects of serial levosimendan infusions on left ventricular performance and plasma biomarkers of myocardial injury and neurohumoral and immune activation in patients with advanced heart failure Heart 2006 92 1768 1772 17105880 10.1136/hrt.2006.079707 PMC1861282

