# Supplementary material for: Single-center experience with levosimendan in children undergoing cardiac surgery and in children with decompensated heart failure
Source: BMC Anesthesiol. 2011 Oct 5;11:18. doi: 10.1186/1471-2253-11-18 (PMC3199236; doi:10.1186/1471-2253-11-18)
Supplement: Additional file 1 — Levosimendan questionnaire. A multiple choice questionnaire for physicians. [file 1471-2253-11-18-S1.DOC]

**Levosimendan questionnaire**

For: anesthesiologist__ cardiologist­­__ surgeon__

1. My indication for the use of levosimendan,

a) patients with cardiomyopathy

b) "low cardiac output syndrome" (LCOS) after cardiac surgery

c) heart transplantation

d) sepsis

e) none above, or no evidence of the benefits of levosimendan

2. Levosimendan seems to be most benficial in

a) patients with cardiomyopathy

b) "low cardiac output syndrome"

c) patients after heart transplantation

d) patients with sepsis

e) never

3. I am using levosimendan

a) as the primary intrope

b) when adrenaline, and noradrenaline, alone or combined are not sufficient

c) when the milrinone alone or in combination with the stated above is insufficient to maintain stable hemodynamics

d) never

e) never because it is too expensive

4. Levosimendan administration

a) I always start with the bolus dose of 12 to 24mcg/kg

b) I start with the bolus dose if the patient is not hypotensive

5. Levosimendan is more efficient compared to

a) dopamine

c) dobutamine

b) adrenaline

c) milrinone

6. According to the literature levosimendan in children

a) is found to be safe for use

b) has sufficient evidence for its effectiveness

c) levosimendan can be regarded as an effective treatment in children on the basis of the experimental and adult studies

d) insufficient evidence in the literature to use levosimendan in all patients

7. The best way to use levosimendan in children undergoing cardiac surgery is

a) after anesthesia induction in patients at risk for postoperative LCOS

b) during the weaning from cardiopulmonary bypass and the other inotropes that are insufficient to maintain stable hemodynamics

c) postoperatively in the PICU when the other inotropes are insufficient to maintain stable hemodynamics

8. On the basis of my experience the efficacy of levosimendan in repeated infusions

a) remains as effective as at the first infusion

b) after the first infusion the efficiency of levosimendan declines

9. The adverse events I have observed during levosimendan administration are

a) hypotension in the beginning of the infusion

b) tachycardia in the beginning of the infusion

c) ventricular extrasystolia

d) atrial fibrillation

e) no adverse events

10. Since 2001 when we started using Levosimendan

a) levosimendan treatment has saved the lives of some children when the other treatments have failed

b) levosimendan treatment has saved the lives of dozens children when the other treatments have failed

c) levosimendan treatment has not saved any lives

d) levosimendan treatment has postponed the need for mechanical assist devices in patients with cardiomyopathy

e) mechanical support could have been totally avoided in patients with cardiomyopathy after receiving levosimendan

f) mechanical support could have been totally avoided in children undergoing cardiac surgery after receiving levosimendan

g) I can not recall any children in whom mechanical support could have been totally avoided because of levosimendan administration
